# Polyreactive Monoclonal Autoantibodies in Multiple Sclerosis: Functional Selection from Phage Display Library and Characterization by Deep Sequencing Analysis

**Published:** 2013

**Authors:** Y. A. Lomakin, M. Yu. Zakharova, A. A. Belogurov, N. A. Bykova, M. A. Dronina, A. E. Tupikin, V. D. Knorre, A. N. Boyko, A. V. Favorov, M. R. Kabilov, N. A. Ponomarenko, A. G. Gabibov

**Affiliations:** Shemyakin–Ovchinnikov Institute of Bioorganic Chemistry, Russian Academy of Sciences, 117997, Moscow, Russia; Institute of Gene Biology, Russian Academy of Sciences, 119334, Moscow, Russia; Kharkevich Institute for Information Transmission Problems, Russian Academy of Sciences, 127994, Moscow, Russia; Faculty of Bioengineering and Bioinformatics, Lomonosov Moscow State University, 119991, Moscow, Russia; Institute of Chemical Biology and Fundamental Medicine, Siberian Branch, Russian Academy of Sciences, 630090, Novosibirsk, Russia; Genomics Core Facility, Siberian Branch, Russian Academy of Sciences, 630090, Novosibirsk, Russia; Moscow Multiple Sclerosis Center at the City Hospital #11, 127018, Moscow, Russia; Pirogov Russian National Research Medical University, Department of Fundamental and Clinical Neurology and Neurosurgery, 117997, Moscow, Russia; Vavilov Institute of General Genetics, Russian Academy of Sciences, 119991, Moscow, Russia; Department of Oncology, Division of Biostatistics and Bioinformatics, Johns Hopkins University School of Medicine, 21218, Baltimore, Maryland, USA; State Research Institute of Genetics and Selection of Industrial Microorganisms GosNIIGenetika, 117545, Moscow, Russia; Faculty of Chemistry, Lomonosov Moscow State University, 119991, Moscow, Russia

**Keywords:** Multiple sclerosis, deep sequencing, cross-reactivity, autoreactive B cells, myelin basic protein, viral triggers

## Abstract

Multiple Sclerosis (MS) is a chronic inflammatory demyelinating disease of the central nervous system that primarily affects young and middle-aged people. It is widely accepted that B lymphocyte activation is required for MS progression. Despite the fact that the exact triggering mechanisms of MS remain enigmatic, one may suggest that MS can be induced by viral or bacterial infection in combination with specific genetic and environmental factors. Using deep sequencing and functional selection methodologies we characterized clones of poly- and cross-reactive antibodies that are capable of simultaneous recognition of viral proteins and autoantigens. The latter, in turn, possibly may trigger MS progression through molecular mimicry. It was identified that two cross-reactive antigens are probably recognized by light or heavy chains individually. According to the high structural homology between selected autoantibodies and a number of various antiviral IgGs, we suggest that a wide range of pathogens, instead of a single virus, be regarded as possible triggers of MS.

## INTRODUCTION

Multiple sclerosis (MS) is a chronic inflammatory demyelinating disease of the central nervous system that affects mainly young and middle-aged people at a rate of 3: 10,000. There are more than 2.5 million people with MS all over the world [1]. Thus, MS is the most common demyelinating neuroinflammatory disorder wherein the immune system of a body destroys the myelin sheath of axons for reasons that remain unclear [2]. Social and economic factors are of great importance in this disease due to severe symptoms, including optic neuritis, loss of bowel and bladder control, severe paralysis, and also long duration of the chronic period. In 80% of cases, the disease begins as a relapsing-remitting form, which eventually morphs into the secondary progressive course. Much less frequently (in 20% of cases), the primary progressive form of MS occurs from the very onset [3].

Despite the numerous studies on the etiology of MS, neither the exact cause of its development nor a potential pathogen capable of inducing the disease is known thus far. It is believed that the development of MS requires a predisposition; i.e. the chronic activation of immune cells leading to neuronal damage is possible only under a certain combination of genetic and environmental factors. Genetic screening has identified several candidate genes. *HLA *(human leukocyte antigen) is considered to be the most important of them, since it is associated with the MS region to the greatest extent. Unfortunately, no unambiguous correlations have been identified in this case. Thus, in Northern Europe association between the disease and *HLA*-*DR2 *or HLA-DRB1*15 has been historically established [4, 5], while in other parts of Europe (e.g., Sardinia) the strongest association was determined with HLA-DRB1*0301, HLA-DRB1*0405, and HLADRB1* 1303 [6]. According to other sources, new haplotypes (HLA-DRB1*03, HLA-DRB1*01, HLA-DRB1*10, HLA-DRB1*11, HLA-DRB1*14 and HLA-DRB1*08) have also been found, correlating with the pathology both negatively and positively, but the strength of the effect varied from case to case [7-10]. Nevertheless, increased risk of MS development by the relatives of MS patients was unambiguously identified [11-14]. The risk of MS in first-degree relatives was about 10–25 times higher compared to that of a normal population sample. Association between the *CD40 *gene (rs6074022) and MS has also been identified [15]. A significant genetic determinism of individual response to treatment with many drugs may be evidence of genetic predisposition. For example, the pharmacogenomic studies of MS have revealed a significant role for a number of polymorphic variants of genes (*CCR5, DRB1, IFNG, TGFB1, IFNAR1, IL7RA, *and possibly*, TNF *and *CTLA4*) in response to the administration of Copaxone [16]. Epidemiological studies, in turn, have identified several risk factors for MS, including bacterial and viral infections, climatic conditions, and smoking.

Although the cause of MS remains unknown, the disease is always accompanied by similar processes, such as activation and increase in immune cell number in the CN S, which further leads to demyelination, axonic/ neuronal damage, and death of oligodendrocytes; these are significant symptoms of MS [17]. At the initial stages of studying MS, the major role in the development of the disease was attributed to T lymphocytes. But now we can confidently assert that activation of B cells is required for pathology development. In addition to producing pathogenic autoantibodies, B lymphocytes are also active antigen-presenting (APC) and cytokineproducing cells [18]. The list of potential autoantigens in MS is constantly expanding and includes various proteins associated with the oligodendrocyte membrane. The emphasis is on the myelin basic protein (MBP), proteolipid protein (PLP1), and myelin-oligodendrocytal glycoprotein (MOG). Moreover, the catalytic antibodies to MBP, which not only bind but also hydrolyze it, were found in the serum of MS patients and SJL mice with experimental autoimmune encephalomyelitis (MS model) [19-21].

Thus, detection of a foreign (e.g., viral) antigen capable of inducing the production of autoantibodies to components of the myelin sheath, and analysis of the structure of these antibodies may be extremely promising for understanding the mechanisms of the disease and developing new approaches to MS treatment and diagnostics.

To date, there is no medical protocol that would allow complete curing of MS patients. Betaferon, which lowers the inflammation level in the CN S [22], and Copaxone, which also reduces the exacerbation frequency [23], are the most commonly used agents in MS patients. Vaccines aimed at eliminating autoreactive B cells are being designed; the already certified drug product Rituximab (a monoclonal antibody that eliminates all B cells) is the best known among them. There are also pilot projects focused on specific elimination [24] or suppression [25] of autoreactive B cells that are precisely known to be pathogenic.

A scFv phage display library has been constructed in our laboratory on the basis of genetic material from MS patients [26]. A series of monoclonal antibodies binding MBP have been selected and characterized. *In vitro *cross-reactivity between MBP and latent membrane protein 1 (LMP-1 protein) of the Epstein-Barr virus (EBV) has been shown for one of these antibodies. A series of papers on the possible viral induction of the disease by molecular mimicry have recently been published [27-29]; the results were further evidence of the triggering role of EBV. In this work, we set out to determine how unique the formation of cross-reactive autoantibodies to MBP and LMP-1 is. To do this, we purposefully obtained cross-reactive clones by successive enrichment of the library on these two antigens. An analysis of their structure and germlines revealed the high diversity of these cross-reactive clones, which have the potential of inducing MS. It is interesting to note that most of the obtained antibodies are highly homologous to the antibodies to proteins of other pathogens, which may be regarded as an extension of the list of potential MS triggers.

## EXPERIMENTAL

**Reagents**

Agar, tryptone, yeast extract (Difco, UK), mono- and dibasic sodium phosphate, sodium chloride, bovine serum albumin, fraction V (BSA), ethidium bromide, β-mercaptoethanol (Sigma, USA), acrylamide, N`,N`- methylenebisacrylamide, sodium dodecyl sulfate (SDS), urea, Hybond C extra nitrocellulose membrane (Amersham, USA), NP-40 surfactant glycine, isopropyl-β-Dthiogalactopyranoside (IPTG) (Fermentas, Lithuania), TMB (tetramethylbenzidine) solution (ZAO “NBO Immunotech”, Russia) were used. The other reagents were produced in Russia and were of ultrapure grade.

**Enzymes**

Thermostable DNA-dependent DNA polymerase, alkaline phosphatase, Rapid DNA Ligation kit (Fermentas, Lithuania), restriction endonucleases and the corresponding standard buffer solutions (Fermentas, Lithuania), deoxyribonuclease I (Biozyme Laboratories Ltd., USA), trypsin, lysozyme (Merck, Germany) DNA fragment size markers and molecular weight markers: GeneRuler^TM^ 50 bp DNA Ladder GeneRuler^TM^ 1 kb DNA Ladder, Protein Molecular Weight Marker 14.4–116.0 kDa, Prestained Protein Molecular Weight Marker 19.0- 118.0 kDa (Fermentas, Lithuania) low molecular weight marker 2.5–16.9 kDa (Amersham, USA).

**Antibodies**

Antibodies to c-myc epitope produced by C-MYC hybridoma antibodies to 3-flag epitope conjugated to horseradish peroxidase (Sigma, USA) antibodies to M13 phage envelope protein conjugated and nonconjugated to horseradish peroxidase (GE Healthcare, USA).

**Protein expression and purification**

Preparations of purified bovine MBP and recombinant human MOG (30–147 a.a.) were done according to the previously published procedure [21]. Recombinant LMP-1 was expressed in HEK293 eukaryotic cells. HEK293 cells were transfected with pBudCE 1.4/EF/ LMP1-FLAG-His-Strep plasmid using unifectin-56. Cells were lysed in a RIPA buffer with 1 M urea and inhibitor cocktail (Roche, Germany) overnight at 4°C under continuous stirring. LMP-1 was purified from the lysate using anti-FLAG agarose in agreement with the protocol. The N-and C-terminal domains of LMP-1 were purified using affinity chromatography on Talon resin (Clontech, USA) and then using the MonoQ sepharose column (Amersham).

**Enrichment of the library**

The scFv phage display library derived from the peripheral blood lymphocytes of MS patients was described previously [26]. The enrichment was carried out according to the procedure (Tomlison protocol; Source BioScience, http://www.lifesciences.sourcebioscience. com) with minor modifications. 10 μg/ml of antigen (MBP, MOG , LMP-1) was absorbed on immuno tubes (Immuno Tubes maxisorp, Nunc, Germany) in a carbonate buffer (pH 9.2). Two rounds of biopanning were carried out for each antigen. Two additional rounds using MBP as an antigen were conducted in the case of double enrichment for LMP-1/MBP.

**ELISA**

Antigen diluted in a 0.1 M carbonate buffer to a concentration of 5 μg/ml was adsorbed on polystyrene plates (MaxiSorp, Denmark) overnight at 4°C. The next day, after removal of the antigen, the wells were washed 3 times with a PBS buffer with 0.1% Tween 20. Nonspecific binding regions were blocked with a 3% BSA solution in PBS, pH 7.2 (37°C, 1 h). Thereafter, the wells were washed thrice with the PBS buffer with 0.1% Tween 20 again and then incubated for 1 h at 37°C with the second layer reactants in the PBS buffer with 0.1% Tween 20. Washing with the PBS buffer with 0.1% Tween 20 was performed three times after each incubation. The antibodies of the last layer were conjugated to horseradish peroxidase. Development was conducted using the proprietary reagent TMB (ZAO “NBO Immunotech“, Russia); the reaction was quenched with 10% H_3_PO_4_. The absorbance (A_450_) was measured using a Varioscan Flash microplate reader (Thermo Scientific, USA).

**Deep sequencing of genes of the V_H_/V_L_ variable regions from phage display libraries**

The original MS phage display library and four sublibraries enriched for different antigens (MBP, MOG, LMP-1, double enrichment for LMP-1/MBP) were amplified in the TG-1 cells of *Escherichia coli*. PCR was performed using Phusion Hot Start II High-Fidelity DNA Polymerase (Fermentas, Lithuania). The reaction mixture contained 5 ng of DNA plasmid as a template and 10 pmol of the flanking primers. PCR products were purified using a GeneJET Gel Extraction Kit (Thermo Scientific, USA) and ligated with NE BNext Multiplex Oligos adapters (New England Biolabs, UK) using the NE BNext Ultra DNA Library Prep Kit for Illumina (New England Biolabs, UK). After the samples had been prepared, the libraries were analyzed both quantitatively using Qubit (Invitrogen) and qualitatively using the 2100 Bioanalyzer (Agilent Technologies). The libraries were normalized to a concentration of 10 nM based on counting and mixed at an equimolar ratio. Amplification of the samples was carried out according to the protocol (Illumina) using MiSeq with the Reagent Kit v2 ( 2 X 250). Merging and alignment of the related readings was carried out based on GW CLC Bio. The characteristics of the antibodies were determined immediately after the deep sequencing using the IMGT/HighV-QUE ST online resource [30].

**Filter parameters for analyzing the occurrence of the hypervariable regions**

The results of the alignment of the sequences obtained using deep sequencing after the analysis using the IMGT/V-QUE ST software [ 31] were filtered by the following criteria: the “Functionality” field of the alignment should be “productive“ (the antibody sequence should be evaluated by the program as productive); the identity of V-gene alleles and germline from the IMGT database should be 70 % or higher; and the sequences of the light chains identified by the IMGT/V-QUE ST program as heavy chains were not considered.

## RESULTS AND DISCUSSION

**Fig. 1:**
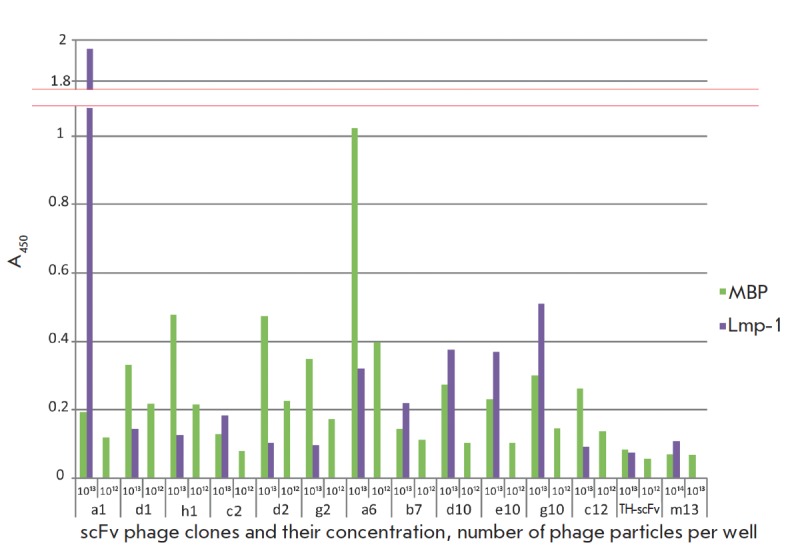
Monoclonal phage ELISA of MBP (green bars) and lmp1 (violet bars) binding by the antibodies under study. The bacteriophage M13K07 (m13) and bacteriophage exposing on its surface scFv towards thyroglobulin (TH-scFv) were used as negative controls

We analyzed the structures and representation of the antibodies selected for the major MS autoantigens – MBP and MOG, as well as viral protein LMP-1, which was previously shown to be a potential trigger of MS [26]. To this end, two rounds of enrichment for MOG and LMP-1 (the enrichment for MBP was conducted previously [26]), and two consecutive rounds of biopanning for LMP-1 and then two rounds for MBP to find cross-reactive antibodies, were conducted. All of the enrichment was carried out under the control of polyclonal ELISA. After the selection, the resulting scFvs as a component of phage particles were analyzed using monoclonal ELISA. A clone was considered positive if the signal of its binding to one or two antigens in ELISA exceeded by threefold the signal of the M13K07 phage used as a negative control (a titer of 1013 phage particles/well). The ELISA results for the most promising clones capable of binding both MBP and LMP-1 are shown in *Fig. 1*. The ability of these phage clones to bind both antigens was confirmed by at least three independent ELISA tests.

As a result, several phage clones carrying scFvs, which most efficiently bound to LMP-1 or LMP-1/MBP, were selected. The Table lists the data on the relative strength of binding of phage clones to the analyzed antigens, as well as the amino acid sequences of their hypervariable regions and related germlines. Several interesting patterns can be noticed by comparing the structures of the resulting scFvs: (1) multiple selection of individual light chains both on LMP-1 and on two antigens; (2) occurrence of identical light chains both in free form and as part of scFv – c12 and b3 LL, g3 LL, d4 LL clones. Selection of specific light chains both in free form for the selection on LMP-1 (b3 LL, g3 LL, d4 LL) and as part of scFv in obtaining crossreactive clones (c12) may indicate their essential role in LMP-1 binding, which has been used as the first antigen in double selection. Meanwhile, their combination with a heavy chain is probably a necessity for further binding of MBP; i.e., in this case binding to two antigens and potential cross-reactivity is determined by recognition of the related antigen by heavy and light chains separately. Another interesting fact is that the relative strength of binding of anti-LMP-1-antibodies to the related antigen is much higher than that of binding to both antigens of potentially cross-reactive anti-LMP-1/-anti-MBP-antibodies (Table). These observations may reflect the natural situation occurring during the development of MS, when the primarily formed antibodies to some pathogen (e.g. EBV) can later interact with MBP as they enter the central nervous system (if the blood-brain barrier (BBB) is damaged), causing degradation of the myelin sheath. Apparently, the ability of these antibodies to exhibit potential polyreactivity, albeit at weak binding, is preferable over high specificity with strong affinity.

**Table:** ELISA of scFvs selected after two rounds of biopanning for respective antigens as indicated Note. LL in the scFv name indicates that this clone was selected upon enrichment of library for LMP-1. Relative binding was calculated as a ratio between the observed signal demonstrated by selected scFv phage clone divided by the signal of the negative control (M13K07 phage) added at the same amount. Symbols: exceedence over background signal more than 1.5-fold (±), threefold (+), sixfold (++), ninefold (+++), not determined (ND). All experiments were performed in triplicates.

Selection for LMP-1 / MBP								Binding to LMP-1	Binding to MBP
Clone	V_h_	D_h_	J_h_	H-CDR3	V_L_	J_L_	L-CDR3		
b7	IGHV1-2*04	IGHD1-26*01	IGHJ4*02	VRGSTYSPSGYFEY	IGKV5-2*01	IGKJ2*01	LQHDNFP	+	++
g10	IGHV1-3*01	IGHD6-13*01	IGHJ4*02	ARIFEGLSGIAAPFDY	IGKV4-1*01	IGKJ4*01	QQYFSSPLT	+	±
h1	IGHV1-8*01	IGHD4-17*01	IGHJ6*03	AREVSDYSDYGDVYYMDV	IGKV3-20*01	IGKJ5*01	QQYCC SPIT	±	±
e11	IGHV1-8*01	IGHD4-17*01	IGHJ6*03	AREVSDYSDYGDVYYMDV	IGLV1-44*01	IGLJ3*02	AAWDGSLNGP	±	±
h11	IGHV1-18*04	IGHD3-3*02	IGHJ2*01	ARREEGLYTTSPGYFGV	IGLV3-21*03	IGLJ7*02	RVWDKQTVSRSG	±	+
e12	IGHV1-46*01	IGHD4-11*01	IGHJ6*02	ARRGFDY	IGKV1-33*01	IGKJ1*01	LQFYEFPYT	±	±
c11	IGHV1-46*03	IGHD5-12*01	IGHJ6*03	AKDLRPRDIGDMDV	IGKV1-39*01	IGKJ5*01	QQSYSSP	±	+
c12	IGHV1-69*06	IGHD1-26*01	IGHJ6*02	ARCGILRSHYFYGMDV	IGLV1-47*01	IGLJ7*01	AAWDDSLSG	±	+
a6	IGHV3-7*01	IGHD4-11*01	IGHJ6*02	VRGGLGAGADY	IGLV4-69*01	IGLJ7*01	QTWGTGI	+	++
c3	IGHV4-b*01	IGHD2-21*01	IGHJ5*01	AGLTQSSHNDAN	IGKV2-30*01	IGKJ1*01	MQATHWP	±	+
f11 (=e1 LL)	-	-	-	-	IGLV1-47*01	IGLJ3*02	VAWDDNLSG	±	±
c2	-	-	-	-	IGLV3-1*01	IGLJ7*01	AAWDDSLNGPV	±	±
d1	-	-	-	-	IGLV6-57*01	IGLJ7*01	QSYNTSTLI	±	±
a1	-	-	-	-	IGLV10-54*01	IGLJ3*02	SVWDSSLSA	±	±
Selection for LMP-1									
h4 LL	IGHV3-23*01	IGHD6-13*01	IGHJ2*01	AKDIAAAATTPEY	IGKV3-11*01	IGKJ5*01	QQRSNWPPT	+++	ND
c12 LL	IGHV5-51*01	IGHD4-17*01	IGHJ4*03	ARFYDSTGSCDY	IGKV1D-33*01	IGKJ2*02	SIIQXKFPLXC	+++	ND
d4 LL, g3 LL, b3 LL	-	-	-	-	IGLV1-47*01	IGLJ7*01	AAWDDSLSG	+++	ND
e1 LL (=f11)	-	-	-	-	IGLV1-47*01	IGLJ3*02	VAWDDNLSG	+++	ND
d2 LL (=a1)	-	-	-	-	IGLV10-54*01	IGLJ3*02	SVWDSSLSA	+++	ND

**Fig. 2:**
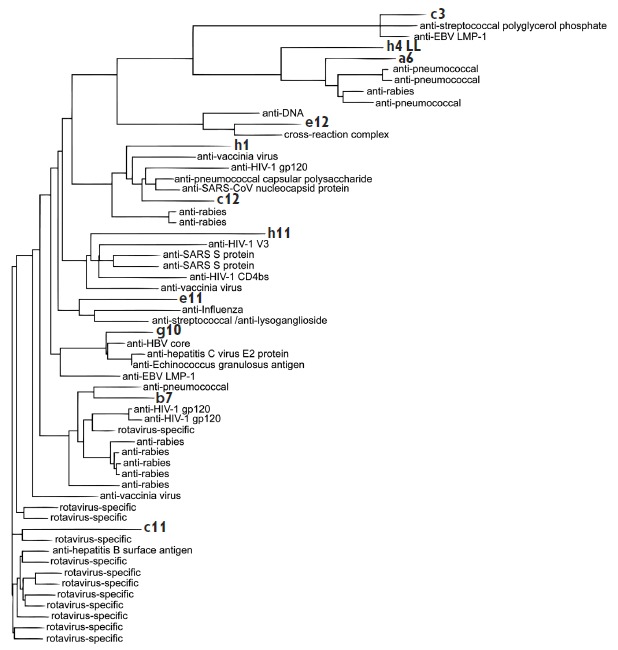
Homology of the selected heavy chains with antiviral antibodies as indicated. The scFvs selected in the current study are shown in bold

**Fig. 3:**
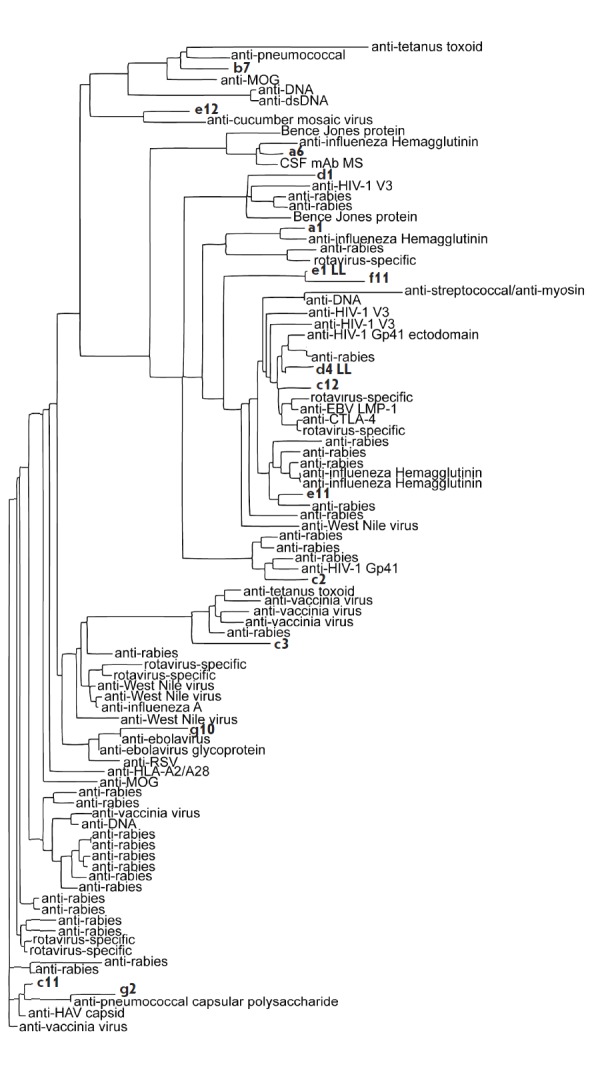
Homology of the selected light chains with antiviral antibodies as indicated. The scFvs selected in the current study are shown in bold

Homology search among the selected monoclonal antibodies was performed for the amino acid sequences using the Protein Data Bank (pdb), UniProtKB / Swiss- Prot (swissprot) databases and protein BLAST software. Figures 2 and 3 show data on the relative homology between the structures of the resulting antibodies and immunoglobulins specific to different viral and bacterial proteins. A high level of similarity between the obtained antibodies and a series of pathogen-specific antibodies (against the influenza virus, West Nile virus, rabies virus, rotavirus, pneumococcus, streptococcus, etc.) was revealed both for heavy and light chains. There is also a high level of structural similarity between the obtained antibodies and antibodies from CSF in MS, anti-MOG, anti-CD152 (cytotoxic lymphocyte antigen 4), and antibodies to the Bence-Jones protein. The data on the homology of the heavy chains structures of cross-reactive c3 antibody and the anti- LMP-1 antibody (gb | ABA55010.1 – 91% homology), as well as cross-reactive g10 and anti-LMP-1 antibody (gb | ABA55014.1 – 86% homology), are of special interest, because it possibly confirms the accuracy of the performed biopanning. As for the light chains, the high homology of the b7 antibody and MOG-specific antibody (gb | AAY15116.1 – 90% homology) may be indicative of the polyreactivity of the selected antibody, while the similarity of a6 and the antibody from the CSF of a MS patient (gb | AAS21063.1 – 94% homology) may confirm the autoimmune nature of the selected antibody. In our opinion, the high proviral homology of the antibodies capable of MBP binding indicates that many viral proteins can act as the primary target for these antibodies. Thus, along with genetic and environmental factors, for MS induction and activation of pathogenic B cells, not a single specific exogenous pathogen is essential, but rather the ability of this pathogen to recruit the immune cells into the central nervous system along with its own penetration, which eventually causes “multiple and disorderly” activation of such cells. In other words, the antibody-secreting cells activated in the peripheral lymph nodes migrate through the damaged BBB. Thus, the primary antibodies to viral antigens interact with their own cross-reactive auto-antigens in the CN S, causing local inflammation and further development of the disease.

**Fig. 4:**
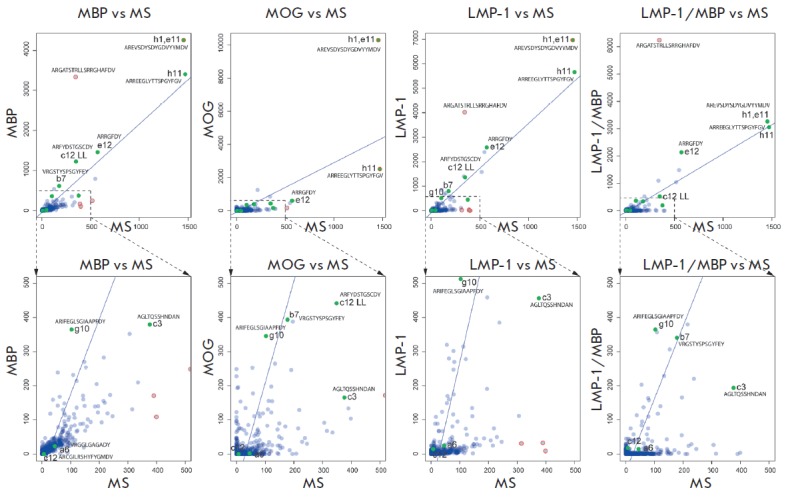
Occurrence of the CDR3 of heavy chain in enriched sublibraries as compared to the initial MS library. Each circle indicates unique CDR3 with the number of reads for this CDR3 in MS library (X axis) and in respective enriched sublibrary (Y axis). For each pair of libraries, the regression and the outlier analysis were done using ‘car’ R package (outliers are colored in red). The functionally selected monoclones are shown in green with indication of their code according to the Table. Sequences of the H-CDR3 for the most interesting clones are indicated

**Fig. 5:**
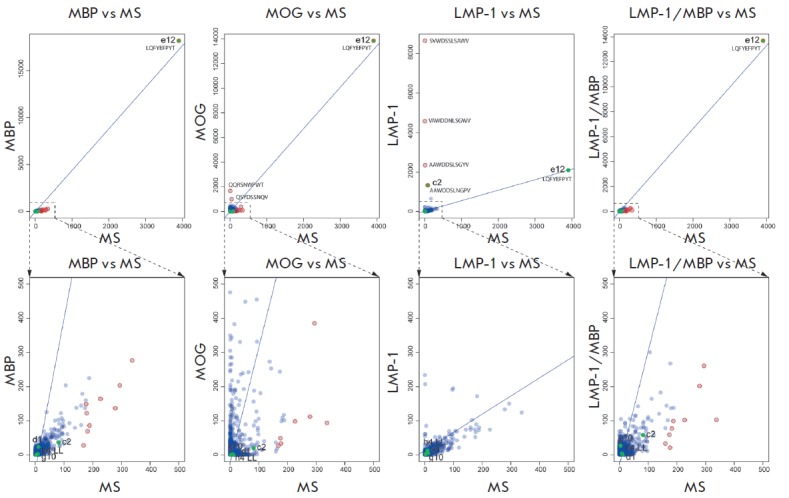
Occurrence of the CDR3 of light chain in enriched sublibraries as compared to the initial MS library. Each circle indicates a unique CDR3 with the number of reads for this CDR3 in MS library (X axis) and in the respective enriched sublibrary (Y axis). For each pair of libraries, the regression and the outlier analysis were done using the ‘car’ R package (outliers are colored in red). The functionally selected monoclones are shown in green with indication of their code according to the Table. Sequences of the L-CDR3 for the most interesting clones are indicated

Deep sequencing of enriched libraries was carried out to evaluate the results of the selection of antibodies to the desired antigens. About 100,000 sequences from each library (50,000 for heavy and light chains, respectively) were identified using the Illumina MiSeq equipment. Further analysis of the heavy and light chains of the selected antibodies for the occurrence of CDR3 was carried out; the relative charges of the most effectively selected CDR3 were determined. To that end, the sequences obtained using deep sequencing were aligned with those of antibodies from the IMGT database [32] using the IMGT/V-QUE ST software [31]. The results of the alignments were then filtered to get rid of artifacts. Only the filtered sequencing results were further analyzed. A comparative analysis was applied to the representation of different CDR3 in the enriched libraries as compared to the initial MS library; the total number of sequences carrying CDR3 was regarded as a measure of CDR3 representation (Figs. 4, 5). Figures 4, 5 also show the representation of CDR3 antibodies obtained using functional selection (Table). The outlier points located above the regression line correspond to positive selection on the given CDR3 (it predominates in this selection compared to the other CDR3), while the outlier points below the line correspond to negative selection. We were primarily interested in the positive outliers, since they were the first candidates for functionally important CDR3 in each selection. As expected, most clones selected after the functional selection using monoclonal phage ELISA were predominant among the CDR3 that were prevailing according to their occurrence. Moreover, it is clear that clones h1 and e11 carry a heavy-chain CDR3 with increased polyreactivity, since the frequency of its occurrence is increased in all four enriched libraries compared to the original one. CDR3 of clone h11 was amplified in the MBP, LMP-1, and LMP-1/MBP libraries, which can characterize it as part of the cross-reactive paratope for two common epitopes in MBP and LMP-1. On the other hand, an unusual situation occurred when the ARGATSTR LLSRR GHAFDV sequence underwent explicit selection for binding of MBP and LMP-1 in the analysis of the occurrence of H-CDR3, but no antibodies with such CDR3 were obtained using monoclonal phage ELISA. This fact could be attributed to the limited number of clones analyzed by phage ELISA. This situation may also result from the low affinity of the specific phage clone for two antigens in monoclonal ELISA, whereby this clone was not selected for further analysis. However, in reality it quantitatively passed selection for two antigens. In any case, further analysis of clones with similar hypervariable regions may help clarify the cross-reactivity problem. Among the light chains, scFv e12 had potentially increased cross-reactivity between MBP and MOG. It was also effectively selected in the LMP-1/MBP library after enrichment for MBP, although no effective selection for this CDR3 was observed upon enrichment for LMP-1.

**Fig. 6:**
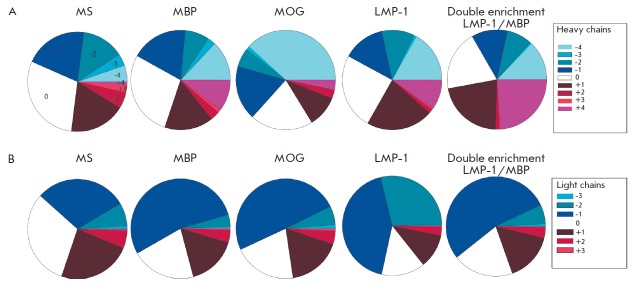
Distribution of the CDR3 net charge in sublibraries enriched for different antigens for heavy (A) and light (B) chains

Interaction between two proteins occurs largely due to the charge in the contact area. Since CDR3 plays the most important role in the formation of an antibody binding site, we decided to evaluate the contribution of electrostatic interactions in this region to the binding specificity in the selection on different antigens, as well as in the selection of cross-reactive clones. We determined the occurrence frequency of CDR3 with different charges for heavy and light chains (Fig. 6), taking into account the number of readings for each sequence. It may be noted that in the library enriched for MOG, the amount of neutrally charged CDR3 of the heavy chain fell almost by a third, and the total amount of CDR3 with a high negative net charge (–4 and higher) increased sevenfold. This is mainly due to the reduction in the positive charge (+1). A shift of the net charge to a higher charge, either positive (+4) or negative (–4), was observed in the case of selection for the other antigens. For the light chains, a decrease in the amount of neutral and an increase in the amount of weekly negative CDR3 (–1 for MBP, MOG and double enrichment LMP-1/MBP and –1–2 for LMP-1) was observed. Thus, a conclusion can be drawn that although the library of antibodies of MS patients (MS in Fig. 6), which to some extent represents the distribution of the antibodies in the patient’s body, is originally dominated by immunoglobulins with a neutral CDR3, the tendency to be autoreactive is mainly characteristic of antibodies with charged residues in the antigen binding sites. Notably, heavy chains demonstrated a more significant charge shift towards the extreme values in absolute magnitude, both positive and negative, compared to light chains. This may be indicative of the more active participation of the heavy chain in the formation of a binding site.

## CONCLUSIONS

We obtained a panel of antibodies to several autoantigens in MS patients, as well as a set of cross-reactive antibodies binding both to the Epstein-Barr virus protein and to the structural unit of the myelin sheath (MBP). The high homology of the antibodies to selected autoantigens and viral or bacterial pathogens may attest to the participation of several viruses in the development of MS. Polyreactivity of autoantibodies in MS patients can be due to the combination of two chains, a heavy and light one, each of them being largely responsible for binding to its own antigen. In the case of sequential selection for LMP- 1 and MBP conducted in this work, the antibody light chain is probably responsible for the binding to LMP- 1, whereas combination with the heavy chain leads to the formation of a full-featured cross-reactive antibody binding both to LMP-1 and MBP. An increase in charged CDR3 is typical of autoantibodies specific for the studied MS autoantigens (MBP, MOG) and a potential viral MS trigger (LMP-1).

## Abbreviations

MS: multiple sclerosis
EBV: Epstein-Barr virus
MBP: myelin basic protein
LMP-1: latent membrane protein 1
MOG: myelin oligodendrocyte glycoprotein
ELISA: enzyme-linked immunosorbent assay
CNS: central nervous system
CSF: cerebrospinal fluid
BBB: blood-brain barrier
